# Complete Nucleotide Sequence of Plasmids of Two *Escherichia coli* Strains Carrying *bla*_NDM–__5_ and *bla*_NDM__–__5_ and *bla*_OXA__–__181_ From the Same Patient

**DOI:** 10.3389/fmicb.2019.03095

**Published:** 2020-01-21

**Authors:** Vittoria Mattioni Marchetti, Ibrahim Bitar, Alessandra Mercato, Elisabetta Nucleo, Annalisa Bonomini, Palmino Pedroni, Jaroslav Hrabak, Roberta Migliavacca

**Affiliations:** ^1^Department of Clinical Surgical Diagnostic and Pediatric Sciences, Unit of Microbiology and Clinical Microbiology, University of Pavia, Pavia, Italy; ^2^Department of Microbiology, Faculty of Medicine in Pilsen, University Hospital in Pilsen, Charles University, Pilsen, Czechia; ^3^Biomedical Center, Faculty of Medicine in Pilsen, Charles University, Pilsen, Czechia; ^4^Clinical Microbiology Laboratory, “AO Desenzano del Garda,” Brescia, Italy

**Keywords:** *E. coli*, NDM-5, OXA-181, CMY-42, WGS, plasmids

## Abstract

Aim of this study was to genetically characterize two carbapenemase-producing *Escherichia coli* strains obtained from a pediatric patient affected by diarrhea, expressing OXA-181 and/or NDM-5 type enzymes. The above microorganisms were collected in the same Desenzano hospital (Northern Italy) where the *bla*_NDM–__5_ gene was detected for the first time in Italy 3 years ago. One strain (5P), belonged to sequence type ST405/ST477 (according to Pasture/Oxford schemes) and serotype O102:H6. It was characterized by a 130562 bp multi-replicon plasmid IncFII/IncFIA/IncFIB (pVSI_NDM-5) enclosing two main antibiotic resistance islands: (i) ARI-I, 10030 bp in size, carried genes coding for β-lactam- (*bla*_OXA–__1_, *bla*_CTX–M–__15_), fluoroquinolone/aminoglycoside- (*aac(6′)-lb-cr*) and phenicol- resistance (*catB3*), (ii) ARI-II, 15326 bp in size, carried genes coding for sulfonamide- (*sul1*), β-lactam- (*bla*_NDM–__5_, *bla*_TEM–__1__B_), phenicol- (*catB3*), trimethoprim- (*dfrA17*), antiseptic- (*qacE*Δ*1*), and aminoglycoside- (*aadA5*, *rmtB*) resistance. The other isolate (5M), belonged to sequence type ST2659/ST759 and serotype O50/02:H18, and carried four plasmids: a 153866 bp multi-replicon IncFII/IncFIA/IncFIB (pISV_IncFII_NDM-5), an 89866 bp IncI1 plasmid, a 51480 bp IncX3 plasmid (pISV_IncX3_OXA181), and a 41143 bp IncI plasmid (pISV_IncI_CMY-42). pISV_IncFII_NDM-5 carried two main antibiotic resistance islands: (i) ARI-III, 12220 bp in size, carried genes coding for β-lactam- (*bla*_OXA–__1_), fluoroquinolone/aminoglycoside- (*aac(6′)-lb-cr*), tetracycline- (*tet(B)*) and phenicol- resistance (*catB3*, *catA1*), and ii) ARI-IV, 26527 bp in size, carried determinants coding for macrolide- (*erm(B)*, *mph(A)*), sulfonamide- (*sul1*), beta-lactam- (*bla*_NDM–__5_, *bla*_TEM–__1__B_), trimethoprim- (*dfrA14*, *dfrA12*), antiseptic- (*qacE*Δ*1*), and aminoglycoside- resistance (*aadA5*). pISV_IncI_CMY-42 harbored the *bla*_CMY–__42_ gene coding for beta-lactam resistance, pISV_IncX3_OXA181 harbored genes encoding fluoroquinolone- (*qnrS1*) and beta-lactams- resistance (*bla*_OXA–__181_). In conclusion, the detection of two different NDM-5 *E. coli* strains from a pediatric patient with a history of travel to the Far East countries strongly highlight an increasing trend and risk of importation from such areas.

## Introduction

Carbapenems are considered as last-resort antibiotics useful for the treatment of infectious diseases caused by multidrug-resistant (MDR) Gram-negative microorganisms; however, the recent increase of carbapenemase-producing *Enterobacteriaceae* (CPE) has limited their therapeutic use in public health (Hoang et al., 2019). NDM is one of the most clinically significant carbapenemases, exhibiting a broad spectrum of activity to all penicillins, cephalosporins, and carbapenems, except aztreonam (Jeon et al., 2015). Since the first report of NDM-1 in 2008, 24 NDM variants have been identified and disseminated worldwide at different extent (Wu et al., 2019). In 2011 NDM-5, which has two amino substitutions (Val88Leu and Met154Leu) compared to NDM-1 (Yang et al., 2015), was reported for the first time in clinical *Escherichia coli* strain isolated in the United Kingdom (Hornsey et al., 2011). Not long after, it has been reported in Japan (Nakano et al., 2014), China (Shen et al., 2018), United States (Mediavilla et al., 2016), South Korea (Park et al., 2016), Lebanon (Dagher et al., 2019). In Italy, NDM-5 was firstly reported in 2017 from an *E. coli* strain isolated from a patient with travel history to Thailand, who was hospitalized in Desenzano del Garda (Bitar et al., 2017). According to ECDC 2018–2019 regional report^1^, outbreaks with involvement of NDM-producers have been increasingly reported from Italy.

OXA-48-like types hydrolyze penicillins at a high level and carbapenems at lower level spearing broad-spectrum cephalosporins and are not inhibited by clavulanic acid, tazobactam, and sulbactam (Poirel et al., 2012). Since the first identification in 2001 in Turkey (Poirel et al., 2004), 11 *bla*_OXA–__48_ gene variants have been reported. Among those, OXA-181 has globally led to a fast spread, causing infections mainly in patients with travel history to the Indian subcontinent (Castanheira et al., 2010, Mairi et al., 2017). The *bla*_OXA–__181_ gene has been detected mostly in *Klebsiella pneumoniae*, with some reports on NDM-5 and OXA-181 co-production in hospitalized patient in South Korea (Cho et al., 2015) and in a patient hospitalized in India then in the United States (Rojas et al., 2017). As suggested in many studies, horizontal transfer of OXA-48-like plasmids between *K. pneumoniae* and commensal *E. coli* can occur in patients initially colonized by hyper-epidemic clones of *K. pneumoniae* (Ortega et al., 2016).

Co-production of NDM and OXA-48-like in *E. coli* was reported in Italy (Monaco et al., 2018), South Korea (Baek et al., 2019), Myanmar (Aung et al., 2018), and Iran (Solgi et al., 2017). The recent dissemination of NDM- and OXA-48-like -producing *E. coli* is usually not related to a single clone but involving multiple sequence types (STs) such as ST410 and ST10 (Pasteur scheme). This sporadic but global dissemination is leading *E. coli* to serve as potential secondary reservoir of such class B and D carbapenemases (Nordmann et al., 2011). Moreover, international travels and migration flows greatly contribute to global spread of carbapenemase producing bacteria.

The aim of this study was to genetically characterize two carbapenemase-producing *E. coli* strains with OXA-181 and/or NDM-5 enzymes and collected form a baby affected by diarrhea; this is a second report from Desenzano del Garda (Brescia, Northern Italy) hospital, where the *bla*_NDM–__5_ was detected for the first time in Italy (in an *E. coli* strain), in 2015.

## Materials and Methods

### Bacterial Isolation

In September 26, 2018, at the Clinical Microbiology Laboratory of Manerbio Hospital (Desenzano, Brescia, Italy) two *E. coli* strains were obtained from a stool specimen of a baby (age between 1 and 5 years old) of Indian origin. The outpatient was suffering from diarrhea and had a travel history to India before being hospitalized in Italy. A Film array gastrointestinal panel (BioFire FilmArray GI Panel, BioMerieux, France) was performed for the evaluation of diarrheagenic *E. coli*, that resulted positive for enteropathogenic *E. coli*. Antibiotic treatment was not initiated. However, patient was treated symptomatically due to diarrhea and discharged after 1 week (October 06, 2018). The two *E. coli* strains (5M and 5P) were carbapenem-resistant and resulted positive for NDM-like for 5P and NDM-like and OXA-48-like production for 5M by Xpert Carba-R test (GeneXpert, Cepheid). Then the isolates were sent to the University of Pavia, where carbapenemase production was confirmed by the KPC/MBL Confirm kit (Rosco Diagnostic) and polymerase chain reaction (PCR) (Balm et al., 2013; Bitar et al., 2017). Finally, the isolates were sent to Charles University in Prague for further genomic characterization.

### Carbapenemase Production Confirmation and Susceptibility Testing

The strain species identification was confirmed using matrix-assisted laser desorption ionization-time of flight mass spectrometry (MALDI-TOF MS) using MALDI Biotyper software (Bruker Daltonics, Bremen, Germany). Carbapenemase production was tested by MALDI-TOF MS meropenem hydrolysis assay (Rotova et al., 2017). Production of different carbapenemases (metallo-beta-lactamase, OXA-48 and KPC) was tested using double-disc synergy test with EDTA, phenylboronic acid test and temocillin disc test (Lee et al., 2003; Doi et al., 2008; Glupczynski et al., 2012), respectively. Antimicrobial susceptibility was performed by AutoScan4 System (Beckman Coulter), tigecycline was evaluated using broth microdilution according to EUCAST guidelines^2^.

### Plasmid Conjugation

Conjugal transfer of resistance determinants was tested in liquid medium using the *E. coli* A15 strain (Rif^*r*^) as recipient. Transconjugants were selected on Mueller-Hinton agar plates containing rifampicin (100 mg/l) and ampicillin (50 mg/l). The presence of *bla*_CMY–__like_, *bla*_NDM–__like_, and *bla*_OXA__48__–like_ in the respective transconjugants were confirmed by PCR.

### Plasmid Size

Plasmid size that carried *bla*_NDM–like_ genes was detected by pulsed-field gel electrophoresis (PFGE) of total DNA digested with S1 nuclease (Promega, Madison, WI, United States; Barton et al., 1995). Then the DNA was transferred to a BrightStar-Plus positively charged nylon membrane (Applied Biosystems, Foster City, CA, United States) and hybridized with digoxigenin- labeled *bla*_NDM__–like_ probe (Paskova et al., 2018).

### Whole-Genome Sequencing and Annotation

The genomic DNA of the two *E. coli* strains was extracted using NucleoSpin Microbial DNA kit (Macherey-Nagel, Germany). Sequel I platform (Pacific Biosciences, Menlo Park, CA, United States) was used for sequencing. Library preparation was done following the microbial multiplexing protocol according to the manufacture instructions for sheared DNA. Shearing was performed using g-tubes (Covaris, United States), and no size selection was done during the library preparations. HGAP4 was used to perform the assemblies of the genomes with minimum seed coverage of 30. ResFinder 3.2^3^ (Zankari et al., 2012), PlasmidFinder^4^ (Carattoli et al., 2014), VirulenceFinder 2.0^5^ (Joensen et al., 2014) ISfinder database^6^, MLST 2.0^7^ (Larsen et al., 2012), and CHTyper 1.0^8^ (Camacho et al., 2009) were utilized to detect resistance genes, plasmid replicon type, virulence genes, mobile elements, multilocus sequence types (STs) (according to Oxford and Pasteur schemes) and FimH/FumC type respectively. Open reading frame (ORF) were predicted using RAST 2.0 (Brettin et al., 2015) with default parameters combined with BLASTP/BLASTN. Comparative genome alignments were performed using the Mauve (version 2.3.1). Gene organization and diagrams were sketched using Inkscape 0.92.4^9^.

### Nucleotide Sequence Accession Numbers

The nucleotide sequences of pISV_IncI_CMY-42, pISV_IncFII_NDM-5, and pVSI_IncFII_NDM-5 plasmids were deposited in the GenBank under the accession numbers MN242251, MN218686, and MN197360, respectively.

## Results

The *E. coli* 5M and 5P strains exhibited clinical resistance to piperacillin, piperacillin-tazobactam, cefotaxime, ceftazidime, aztreonam, meropenem, ertapenem, gentamicin, tetracycline, trimethoprim-sulfamethoxazole, ciprofloxacin, amikacin and chloramphenicol, while remained susceptible to tigecycline and colistin [breakpoints (see text footnote 2)]. Whereas 5P was found to carry a *bla*_NDM–__5_ gene, 5M carried both *bla*_NDM–__5_ and *bla*_OXA–__181_.

The β-lactamase genes were transferable through conjugation in both clinical isolates. The isolate 5P transferred one plasmid (pVSI_NDM-5) to the recipient successfully, while 5M transferred the three plasmids (pISV_InvFII_NDM-5, pISV_IncX3_OXA181, and pISV_IncI_CMY-42) to the recipient (Supplementary Table S1).

**FIGURE 1:**
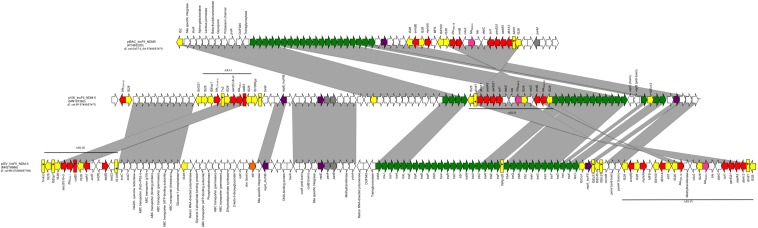
Linear map of pIBA_IncFII_NDM5, pVSI_IncFII_NDM-5, and pISV_IncFII_NDM-5; Arrows show the direction of transcription of ORFs while rectangles show truncated ORFs. Replicons, partitioning genes, mobile elements, conjugal transfer genes, antibiotic resistance, *bla*_NDM–__5_, other remaining genes and stability genes are designated by violet, gray, yellow, green, red, pink white, and orange, respectively. Gray shaded area shows the nucleotide similarity between the three plasmids. Different Antibiotic resistance islands (ARI) are marked by a horizontal black line.

Whole-genome sequencing revealed that 5P belonged to the sequence type ST405/ST477 (according to Pasteur/Oxford schemes), serotype O102:H6 and the CH-Type FumC37/FimH27 (fimbrial adhesion gene f*imH* with allele 27 and fumarate hydratase class II gene *fumC* with allele 37). Moreover, it carried virulence genes encoding *air*, *gad*, *iha* and *sat* coding for enteroaggregative immunoglobulin repeat protein, glutamate decarboxylase, adherence protein and secreted autotransporter toxin respectively. The isolate 5P carried a 130562 bp multi-replicon plasmid IncFII/IncFIA/IncFIB (pVSI_NDM-5). pVSI_NDM-5 plasmid backbone exhibited high similarity scores with the 1336947 bp plasmid pM309-NDM5 carried by an *E. coli* strain isolated in Myanmar in 2019 (AP018833.1; Sugawara et al., 2019) (99.94% sequence identity, 82% query coverage). pVSI_NDM-5 backbone carried regions responsible for replication (*repA*, *repB*, *repE*), conjugative transfer system (*tra*, *finO* genes), maintenance and stability (*parA*, *parB*), toxin-antitoxin (TA) systems (*vapC/vapB*, *ccdA/ccdB*, *pemI/pemK*). Moreover, pVSI_NDM-5 carried two main antibiotic resistance islands (ARI-I and ARI-II); ARI-I, 10030 bp in size, carried genes coding for β-lactam resistance (*bla*_OXA–__1_, *bla*_CTX–M–__15_), fluoroquinolone and aminoglycoside resistance (*aac(6′)-lb-cr*) and phenicol resistance (*catB3*). ARI-I resembled high similarity scores with plasmids from *K. pneumoniae* and *E. coli* (100% sequence similarity and coverage; United States CP023950.1, Denmark MG462728.1) and chromosomal sequences in *Enterobacter cloacae* from Thailand (CP040827.1). While ARI-II, 15326 bp in size, carried genes coding for sulfonamide resistance (*sul1*), β-lactam resistance (*bla*_NDM–__5_, *bla*_TEM–__1__B_), phenicol resistance (*catB3*), trimethoprim resistance (*dfrA17*), antiseptic resistance (*qacE*Δ*1*) and aminoglycoside resistance (*aadA5*, *rmtB*). ARI-II was a composite transposon consisting of two structures: the first structure harbored the *bla*_NDM–__5_ gene with two IS*26* in the same direction on the flanks and another structure harbored *cdu2*, *rmtB* and *bla*_TEM–__1__B_ mediated by another IS*26* on one end in the same direction (Figure 1).

**FIGURE 2:**
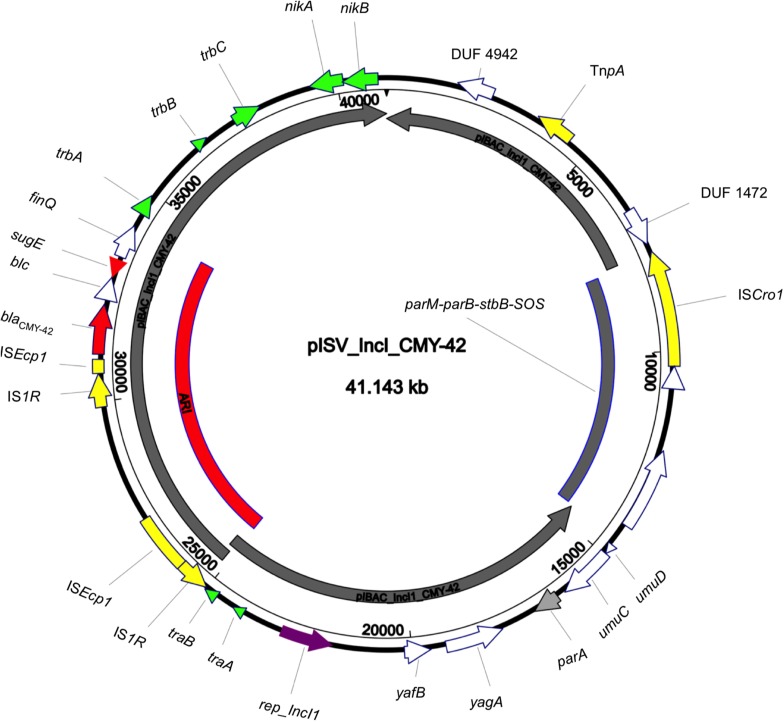
Circular map of pISV_IncI_CMY-42 plasmid compared to pIBAC_IncI_CMY-42 (inner most gray circular box); green arrows represent conjugal transfer system of the plasmid, red arrows represent antibiotic resistance genes, yellow arrows/boxes represent complete/truncated mobile elements, white arrows represent hypothetical proteins, purple arrow represents the replication protein rep_IncI. Further more, the red-arced box represents the plasmid Antibiotic Resistance Island (ARI).

**FIGURE 3:**
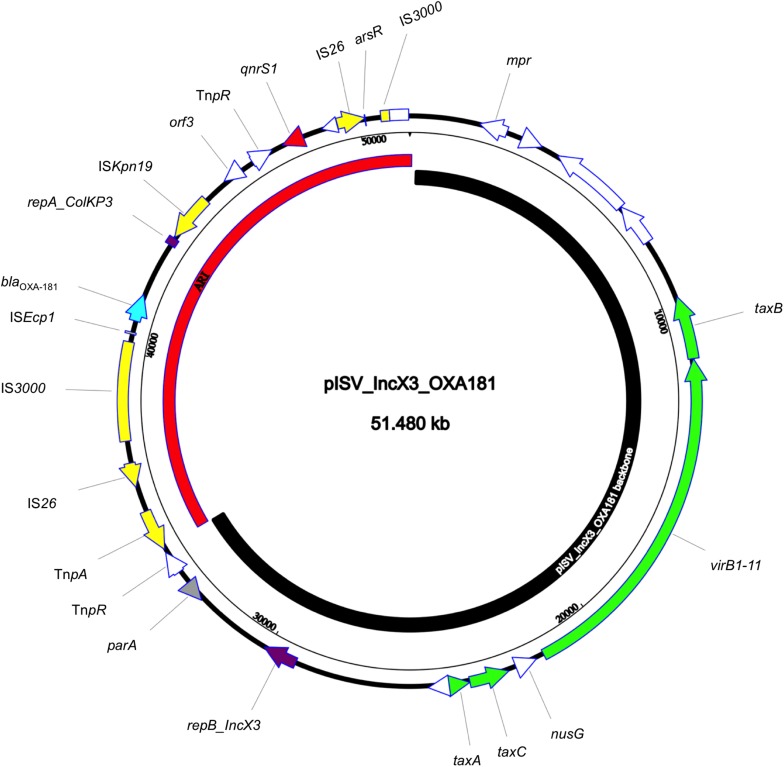
Circular map of pISV_IncX3_OXA181 plasmid; green arrows represent conjugal transfer system of the plasmid, red arrow represents the *qnrS1* gene, blue arrow represents the *bla*_OXA–__181_ gene, yellow arrows/boxes represent complete/truncated mobile elements, white arrows/boxes represent complete/truncated hypothetical proteins, purple arrows/boxes represent complete/truncated replication protein. Further more, the black inner-curved box represents the plasmid backbone while the red one represents the plasmid Antibiotic Resistance Island (ARI).

The isolate 5M belonged to the sequence type ST2659/ST759 serotype O50/02:H18 and the CH-Type FumC26/FimH5 (fimbrial adhesion gene f*imH* with allele 5 and fumarate hydratase class II gene *fumC* with allele 26). Moreover, it carried virulence genes encoding *air*, *gad*, *astA*, *capU* and *eilA* coding for enteroaggregative immunoglobulin repeat protein, glutamate decarboxylase, heat-stable toxin, hexosyltransferase protein and salmonella HilA homologous protein respectively. The isolate 5M carried four plasmids: a 153866 bp multi-replicon plasmid IncFII/IncFIA/IncFIB pISV_IncFII_NDM-5 (Figure 1), a 41143 bp IncI plasmid pISV_IncI_CMY-42 (Figure 2), a 51480 bp IncX3 plasmid (pISV_IncX3_OXA181) (Figure 3) and an 89866 bp IncI1 plasmid (no resistance genes detected). pISV_IncFII_NDM-5 plasmid backbone carried regions responsible for replication (*repA*, *repB*, *repE*), conjugative transfer system (*tra*, *trb*, *finO* genes), maintenance and stability (*parA*, *parB*, *stb*), TA systems (*ccdA/ccdB*, *pemI/pemK*, *doc*). Moreover, pISV_IncFII_NDM-5 carried two main antibiotic resistant islands (ARI-III and ARI-IV); ARI-III, 12220 bp in size, carried genes coding for β-lactam resistance (*bla*_OXA–__1_), fluoroquinolone and aminoglycoside resistance (*aac(6′)-lb-cr*), tetracycline resistance (*tet(B)*) and phenicol resistance (*catB3*, *catA1*). ARI-IV, 26527 bp in size, carried genes coding for macrolide resistance (*erm(B)*, *mph(A)*), sulfonamide resistance (*sul1*), β-lactam resistance (*bla*_NDM–__5_, *bla*_TEM–__1__B_), trimethoprim resistance (*dfrA14*, *dfrA12*), antiseptic resistance (*qacE*Δ*1*) and aminoglycoside resistance (*aadA5*). ARI-IV was found to be in a plasmid isolated in 2017 from an *E. coli* strain in Myanmar pM217_FII (100% sequence similarity, 92% query coverage; AP018147.1) (Sugawara et al., 2017).

The sequence comparison of the two NDM-5 producing plasmids (isolated from 5M and 5P) along with the first reported plasmid producing NDM-5 in Italy revealed that the backbones, and the ARIs, are in some way different. In fact, even though *E. coli* 5P belongs to the same ST405 of the first Italian NDM-5 producing *E. coli* strain (ECDT-2_GA) previously collected from the same hospital and the plasmid belongs to the incompatibility group IncFII (same as the plasmid pIBAC_IncFII_NDM5), the plasmid backbone (*rep*, *tra* and stability genes) were different as well as TA systems (Bitar et al., 2017).

pVSI_IncFII_NDM-5 (carried by 5P) shared most of the regions found in pIBAC_IncFII_NDM5 with more acquired regions as shown in Figure 1. Both of the plasmids shared the ARI-II (in opposite direction); even though pIBAC_IncFII_NDM5 had another segment harboring *erm(B)* and *mph(A)* with 2 IS*25* flanking the peripheries in opposite direction, adjacent to the segment resembling ARI-II. Interestingly, pVSI_IncFII_NDM-5 and pISV_IncFII_NDM-5 shared some regions of the backbone but not with pIBAC_IncFII_NDM5. The ARIs of the two plasmids shared some regions, however pISV_IncFII_NDM-5 ARI-III/IV were larger and carried more resistance genes than pVSI_IncFII_NDM-5 ARI-I/II (except for *bla*_CTX–M–__15_ and *bla*_TEM–__1__B_ found in pVSI_IncFII_NDM-5 only). The re-arrangement of the plasmid backbone in both pISV_IncFII_NDM-5 and pVSI_IncFII_NDM-5), and the excessive presence of insertion sequences (especially IS*26*) in ARIs lead to the formation of complex transposons with similar insertion sequences on the flanking regions (such as in ARI-IV and ARI-II) suggest the possibility of transfer of such regions. Nevertheless, this also suggest that the plasmid itself is not conserved.

pISV_IncI_CMY-42, that harbored the *bla*_CMY–__42_ gene coding for beta-lactam resistance, exhibited high similarity to plasmid pCMY-42 (KY463221.1; 99.48% sequence similarity 93% query coverage) isolated in 2017 from an *E. coli* strain in Italy (Bitar et al., 2017). While pISV_IncX3_OXA181, which harbored genes coding for quinolone resistance (*qnrS1*) and beta-lactam resistance (*bla*_OXA__–__181_), showed 100% sequence similarity and coverage to several plasmids found in NCBI database (AP018837.1 (Sugawara et al., 2019); Myanmar, MG893567.1; China, CP024806; Denmark, MG570092; Lebanon (Bitar et al., 2018), KX894452.1; Germany, KX523903; Czechia).

## Discussion

Here we report a patient with diarrhea that carried two different *E. coli* strains (ST405/ST477 and ST2659/ST759) from the same patient. *E. coli* strain 5P carries one multi-replicon plasmid harboring *bla*_NDM–__5_. The strains were detected by BioFire FilmArray GI panel (BioMerieux), which is based on a multiplex PCR system with a total runtime of about an hour. This method simultaneously tests for 22 targets including Diarrheagenic *E. coli/Shigella*. While the other *E. coli* strain 5M carries three plasmids harboring resistance genes; the first plasmid is a multi-replicon plasmid harboring *bla*_NDM–__5_, the second plasmid harbors *bla*_OXA–__181_ and the third plasmid harbors *bla*_CMY__–__42_. *E. coli* (ST405/ST477) has been reported as an emerging extraintestinal pathogenic *E. coli* (ExPEC), however it is still unknown whether it is a globally dispersed emerging clone, or the disease caused is due to sporadic episodes caused by genetically divergent ST405 isolates (Djordjevic et al., 2018). Furthermore, *E. coli* (ST2659/ST759) has been described so far in two reports from Algeria as a cause of clinical infection (Sassi et al., 2014; Yaici et al., 2016). We hypothesize that the *E. coli* strain (ST2659/ST759) is causing the diarrhea in our patient due to the production of the heat stable toxin (*astA*) which was reported elsewhere as being the cause of diarrhea in patients (Kamjumphol et al., 2019).

The plasmids pVSI_IncFII_NDM-5 and pISV_IncFII_ NDM-5 shared relatively little of its genetic content (Figure 1). The backbones shared: two segments carrying the replicons and partitioning related genes and another segment harboring the ABC transporter system. Moreover, pVSI_IncFII_NDM-5 shared all of its conjugative transfer system (*tra* genes). Nevertheless, pVSI_IncFII_NDM-5 had more *tra* genes and the genes were found in a consecutive manner (complete without interruption) which is not the case in pVSI_IncFII_NDM-5. Moreover, both of ARI-III and ARI-IV of pISV_IncFII_NDM-5 were bigger in size than ARI-I and ARI-II of pVSI_IncFII_NDM-5. Apart of ARI-I being partially present in ARI-III, a part of ARI-II was also present in ARI-IV. The fact that all the ARIs in both plasmids are composite transposons strongly suggests that acquiring/losing segments of ARIs is possible as shown here (Figure 1). Moreover, the position of ARI-II within the conjugal transfer system while ARI-IV (which contain part of the ARI-II) is located after the conjugal transfer system suggests some rearrangements within the plasmid structure.

On the other hand, the comparison of pISV_IncI_ CMY-42 with the plasmid pIBAC_IncI_CMY-42 isolated with pIBAC_IncFII_NDM-5 (Bitar et al., 2017), showed similar plasmid structure except for a small segment that harbored *parM*, *parB*, *stbB* and SOS protein, which was replaced by a segment harboring some hypothetical proteins with the insertion sequence IS*Cro* (Figure 2). pISV_IncX3_OXA181 was the most conserved plasmid among reported IncX3 plasmids harboring the *bla*_OXA–__181_. This successful plasmid that has been reported elsewhere has conserved plasmid backbone as well as conserved ARI (Piazza et al., 2018). This hypothesis is supported by the fact that there is an increasing incidence of this plasmid (100% sequence similarity and query when blasted against the NCBI database) in different geographic settings and this confirms the ease of dissemination of this plasmid (Bitar et al., 2018; Sugawara et al., 2019).

## Conclusion

In conclusion, the detection of two clinically important MDR *E. coli* clones (the pandemic ST405/477 and the toxigenic ST2659/ST759) from the same patient, represents an unusual and concerning picture, despite the increase of NDM outbreaks reports are in Italian area. The presence of genetically different plasmids harboring *bla*_NDM–__5_, suggests that traveling from the Far East countries strongly contributes to the spread of NDM-type class B carbapenemases and suggests the possibility of the presence of hidden reservoirs/healthy carriers in Italy (Pitart et al., 2015; Bitar et al., 2017). The high genomic rearrangement-taking place in these plasmids structure (NDM encoding IncF plasmids) could be explained by the diverse exerted selective pressure in different geographical areas and settings. Finally, the presence of two plasmids harboring *bla*_OXA–__181_ and *bla*_NDM__–__5_, respectively in the same ST2659/ST759 *E. coli* strain confirms the increasing trend in co-replicon existence, which limits the therapeutic options and increase the dissemination risk.

## Data Availability Statement

The nucleotide sequences of pISV_IncI_CMY-42, pISV_IncFII_ NDM-5, and pVSI_IncFII_NDM-5 plasmids were deposited in the GenBank under the accession numbers MN242251, MN218686, and MN197360, respectively.

## Ethics Statement

Ethical review and approval was not required for the study on human participants in accordance with the local legislation and institutional requirements. Written informed consent from the participants’ legal guardian/next of kin was not required to participate in this study in accordance with the national legislation and the institutional requirements.

## Author Contributions

IB, VM, and RM played an important role in interpreting the results and writing the manuscript. AM, EN, AB, PP, and JH helped to acquire the data. IB and VM carried out the experimental work. IB supervised the experiments and revised the final manuscript, which was approved by all authors.

## Conflict of Interest

AB and PP were employed by AO Desenzano del Garda. The remaining authors declare that the research was conducted in the absence of any commercial or financial relationships that could be construed as a potential conflict of interest.

## Footnotes

1 https://ecdc.europa.eu/sites/portal/files/documents/04-Jun-2019-RRA-Carbapenems%2C%20Enterobacteriaceae-Italy.pdf

2 http://www.eucast.org/fileadmin/src/media/PDFs/EUCAST_files/Breakpoint_tables/v_9.0_Breakpoint_Tables.pdf

3 https://cge.cbs.dtu.dk/services/ResFinder/

4 https://cge.cbs.dtu.dk/services/PlasmidFinder/

5 https://cge.cbs.dtu.dk/services/VirulenceFinder/

6 www-is.biotoul.fr/

7 https://cge.cbs.dtu.dk/services/MLST/

8 https://cge.cbs.dtu.dk/services/chtyper/

9 https://inkscape.org/
