# Supplementary material for: Complete Nucleotide Sequence of Plasmids of Two Escherichia coli Strains Carrying blaNDM–5 and blaNDM–5 and blaOXA–181 From the Same Patient
Source: Front Microbiol. 2020 Jan 21;10:3095. doi: 10.3389/fmicb.2019.03095 (PMC6985152; doi:10.3389/fmicb.2019.03095)
Supplement: Supplementary file 1 [file Table_1.DOCX]

Table S1:

Antimicrobial susceptibility profile of conjugation recipient strain A15, donor strains 5M and 5P and the transconjugants A15_5P and A15_5M.

| **Isolate** | **MIC (mg/L)** |  |  |  |  |  |  |  |  |  |  |  |  |  |
| --- | --- | --- | --- | --- | --- | --- | --- | --- | --- | --- | --- | --- | --- | --- |
|  | **AMP** | **GEN** | **AMI** | **CIP** | **CHL** | **TET** | **PIP** | **PPT** | **CTX** | **CAZ** | **MEM** | **ERT** | **NET** | **TOB** |
| **A15** | 1 | 0.12 | 0.5 | ≤0.06 | 4 | 1 | ≤0.5 | 1 | ≤0.06 | ≤0.25 | ≤0.12 | ≤0.5 | ≤0.12 | ≤0.12 |
| **5P** | >128 | >32 | >64 | >8 | 8 | 2 | >128 | >128 | >8 | >16 | 16 | >2 | >16 | >8 |
| **A15_5P** | >128 | >32 | >64 | >8 | 8 | 2 | >128 | >128 | >8 | >16 | 16 | >2 | >16 | >8 |
| **5M** | >128 | >32 | >64 | >8 | >32 | >32 | >128 | >128 | >8 | >16 | 16 | >2 | >16 | >8 |
| **A15_5M** | >128 | >32 | >64 | >8 | >32 | >32 | >128 | >128 | >8 | >16 | 16 | >2 | >16 | >8 |

MIC, minimum inhibitory concentration; AMP, ampicillin; GEN, gentamicin; AMK, amikacin; CIP, ciprofloxacin; CHL, chloramphenicol; TET, tetracycline; PIP, piperacillin; PPT, piperacillin-tazobactam (inhibitor fixed at 4 mg/L); CTX, cefotaxime; CAZ, ceftazidime; MEM, meropenem; ETP, ertapenem; NET, netilmicin; TOB, tobramycin.
